# Complete chloroplast genome of *Hevea benthamiana*, a SALB-resistant relative wild species of rubber tree

**DOI:** 10.1080/23802359.2020.1763868

**Published:** 2020-05-13

**Authors:** Ying-Feng Niu, Yan-Shi Hu, Zi-Yan Liu, Cheng Zheng, Jin Liu

**Affiliations:** a Yunnan Institute of Tropical Crops, Xishuangbanna, China; b Rubber Research Institute, Chinese Academy of Tropical Agriculture Science, Danzhou, China; c Kunming Institute of Botany, Chinese Academy of Sciences, Kunming, China

**Keywords:** *Hevea benthamiana*, chloroplast, genome sequencing

## Abstract

*Hevea benthamiana* is a SALB-resistant wild species of *H. brasiliensis*, the only source of mass production of high quality natural rubber. This study sequenced and analyzed the chloroplast genome of *H. benthamiana*. The chloroplast genome of *H. benthamiana* contains 161,124 bp and consists of 51,495 bp of A (31.96%), 52,022 bp of T (32.29%), 28,915 bp of G (17.95%), and 28,692 bp of C (17.81%). The ring-shaped genome includes four regions: a large single-copy region (LSC, 89,110 bp), a small single copy (SSC, 18,376 bp) region, and two inverted repeat regions (IRs, 26,819 bp). A total of 134 genes were annotated, of which 86 encode proteins; four are pseudogenes; 36 are tRNA genes, and eight are rRNA genes. Phylogenetic analyses showed that *H. benthamiana* is very closely related to *H. Brasiliensis*, this result indicates that *H. benthamiana* is highly valuable for the breeding of SALB-resistant varieties of rubber trees.

*Hevea benthamiana* is a wild species of the rubber tree (*Hevea brasiliensis*), the only source of high quality natural rubber that can be produced on a large scale. The genus *Hevea* consists of 11 species (Gonçalves et al. 1999; Priyadarshan & Gonçalves 2002), but *H. benthamiana* can not only resist South American leaf blight (SALB), which is the most severe disease of rubber trees (Le Guen et al. 2008) caused by *Microcyclus ulei* (Garcia et al. 2011) and responsible for the limited development of rubber plantations in Latin America (Rocha et al. 2011) but it can also hybridize with rubber trees to produce disease-resistant varieties, such as IAN717 (Chee 1976; Lieberei 1986). This is particularly important because the use of resistant cultivars appears to be the most effective way to increase rubber tree plantings in South and Central America, as well as to anticipate an accidental introduction of SALB in Africa or Southeast Asia (Le Guen et al. 2011). *Hevea benthamiana* has been the subject of intensive efforts by rubber tree breeders since it is an important SALB-resistant parent.

Each plant contains at least three sets of relatively independent genetic information, including the nuclear, chloroplast and mitochondrial genomes. The chloroplast genome contains rich genetic information. Therefore, sequencing, assembling and annotating the chloroplast genome is highly important to clarify the genetic background and effective use of species resources. Currently, the nuclear genome of *H. brasiliensis* has been perfectly assembled (Rahman et al. 2013; Lau et al. 2016; Tang et al. 2016; Pootakham et al. 2017; Liu et al. 2020), and the chloroplast genome of *H. brasiliensis* (Tangphatsornruang et al. 2011) and *H. camargoana* (Niu et al. 2020) have also been reported. As a relatively wild species of *H. brasiliensis*, the chloroplast genome of *H. benthamiana* has not yet been reported.

In this study, the chloroplast genome of *H. benthamiana* was sequenced, assembled, and annotated. Bronze-colored young leaves of *H. benthamiana* were collected from The Rubber Tree Germplasm Resource Nursery of the Chinese Academy of Tropical Agriculture Science (N 19°34′31.53″ and E 109°31′17.97″), frozen in liquid nitrogen, and the genomic DNA was extracted using a Rapid Plant Genomic DNA Isolation Kit (Sangon Biotech Shanghai Co. Ltd., China) and stored in an ultra-low temperature specimen library at the Yunnan Institute of Tropical Crops (specimen accession number: YITC-2019-FZ-E-105). The genomic DNA of *H. benthamiana* was sequenced using the Illumina Hi-Seq 2000 platform (http://www.illumina.com, San Diego, CA, USA), and the chloroplast genome was assembled and annotated using CLC Genomics Workbench v3.6 (http://www.clcbio.com) and DOGMA (Wyman et al. 2004). The complete chloroplast sequence of *H. benthamiana* was submitted to GenBank with the accession number of MT333859.

The chloroplast genome of *H. benthamiana* contains 161,124 bp and consists of 51,495 bp of A (31.96%), 52,022 bp of T (32.29%), 28,915 bp of G (17.95%), and 28,692 bp of C (17.81%). Like other plant species, the ring-shaped genome includes four regions: a large single-copy region (LSC, 89,110 bp), a small single copy (SSC, 18,376 bp) region, and two inverted repeat regions (IRs, 26,819 bp). A total of 134 genes were annotated, of which 86 encode proteins; four are pseudogenes, 36 are tRNA genes, and eight are rRNA genes.

The chloroplast genome sequences of 21 species of the Malpighiales order, including *H. benthamiana*, were used for phylogenetic analyses, and *Betula platyphylla*, which belongs to the Fagales order, was used as an outgroup (Figure 1). A phylogenetic tree was constructed using RAxML8.1.5 (https://sco.h-its.org/exelixis/web/software/raxml/index.html) (Alexandros 2006), with a bootstrap value of 1000. Phylogenetic analyses showed that *H. benthamiana* is very closely related to *H. brasiliensis*. This result indicates that *H. benthamiana* is highly valuable for the breeding of SALB-resistant varieties of rubber trees.

**Figure 1.**
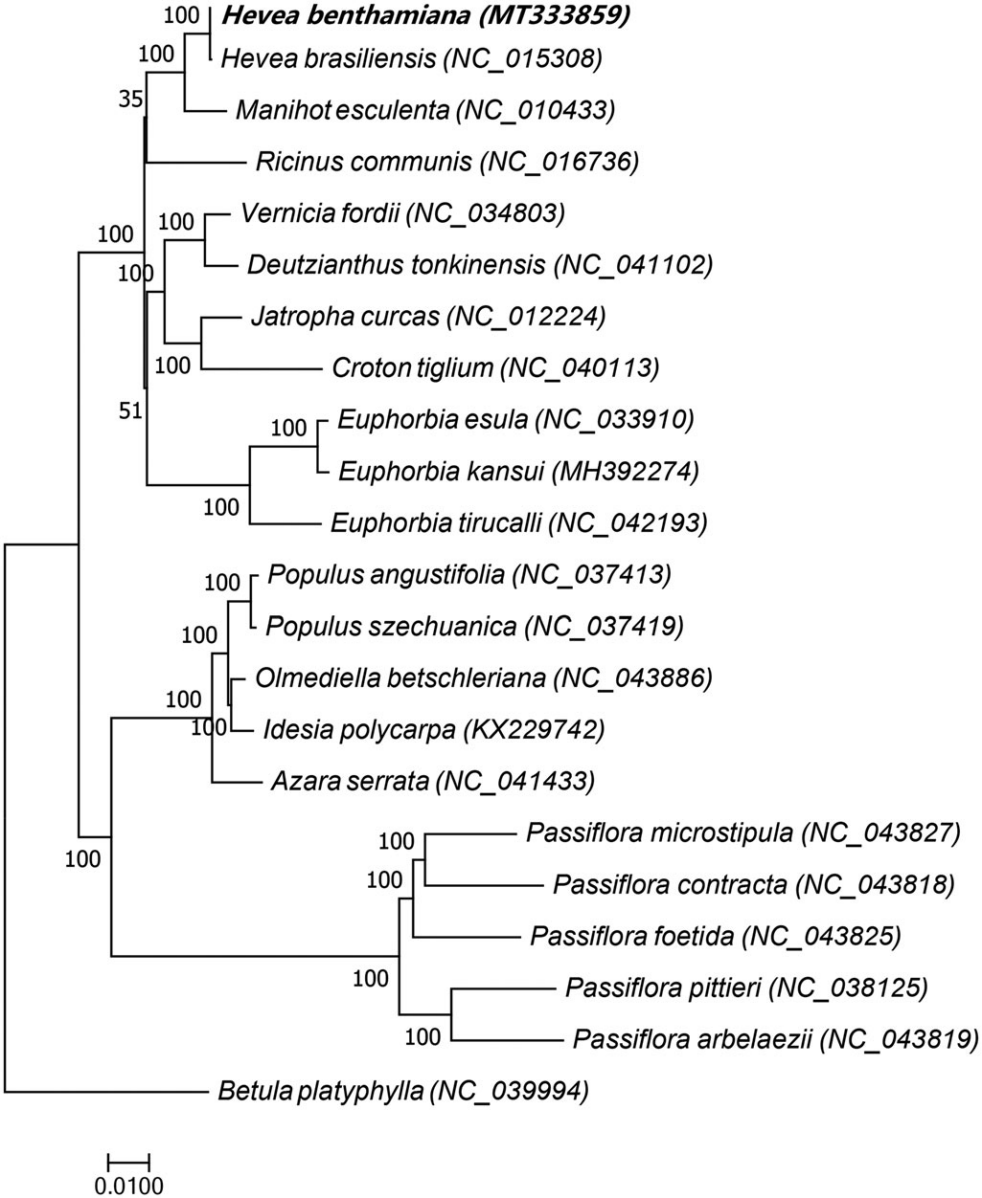
Maximum-likelihood phylogenetic tree based on chloroplast genome sequences of 21 Malpighiales order species, *Betula platyphylla*, which belongs to the Fagales order, was used as an outgroup. The bootstrap value was set to 1000. The species and chloroplast genome accession numbers for tree construction are *Hevea benthamiana* (MT333859), *Hevea brasiliens*is (NC_015308), *Manihot esculenta* (NC_010433), *Ricinus communis* (NC_016736), *Vernicia fordii* (NC_034803), *Deutzianthus tonkinensis* (NC_041102), *Jatropha curcas* (NC_012224), *Croton tiglium* (NC_040113), *Euphorbia esula* (NC_033910), *Euphorbia kansui* (MH392274), *Euphorbia tirucalli* (NC_042193), *Populus angustifolia* (NC_037413), *Populus szechuanica* (NC_037419), *Olmediella betschleriana* (NC_043886), *Idesia polycarpa* (KX229742), *Azara serrata* (NC_041433), *Passiflora microstipula* (NC_043827), *Passiflora contracta* (NC_043818), *Passiflora foetida* (NC_043825), *Passiflora pittieri* (NC_038125), *Passiflora arbelaezii* (NC_043819), and *Betula platyphylla* (NC_039994).

## Data Availability

The data that support the findings of this study are openly available in GenBank at https://www.ncbi.nlm.nih.gov/, reference number MT333859.

## References

[CIT0001] Alexandros S. 2006. RAxML-VI-HPC: maximum likelihood-based phylogenetic analyses with thousands of taxa and mixed models. Bioinformatics. 20:2688–2690. 10.1093/bioinformatics/btl446 16928733

[CIT0002] Chee KH. 1976. Assessing susceptibility of *Hevea* clones to *Microcyclus ulei*. Ann Appl Biol. 84(2):135–145.

[CIT0003] Garcia D, Carels N, Koop DM, de Sousa LA, Andrade Junior SJd, Pujade-Renaud V, Reis Mattos CR, de Mattos Cascardo JC. 2011. EST profiling of resistant and susceptible *Hevea* infected by *Microcyclus ulei*. Physiol Mol Plant Pathol. 76(2):126–136.

[CIT0004] Gonçalves PdS, Fujihara AK, Ortolani AA, Bataglia OC, Bortoletto N, Segnini Junior I. 1999. Origin, variability and domestication of *Hevea*. Pesq Agropec Bras. 34(7):1156–1223.

[CIT0005] Lau N-S, Makita Y, Kawashima M, Taylor TD, Kondo S, Othman AS, Shu-Chien AC, Matsui M. 2016. The rubber tree genome shows expansion of gene family associated with rubber biosynthesis. Sci Rep. 6(1):28594. 27339202 10.1038/srep28594 PMC5008842

[CIT0006] Le Guen V, Garcia D, Doaré F, Mattos CRR, Condina V, Couturier C, Chambon A, Weber C, Espéout S, Seguin M. 2011. A rubber tree’s durable resistance to *Microcyclus ulei* is conferred by a qualitative gene and a major quantitative resistance factor. Tree Genet Genomes. 7(5):877–889.

[CIT0007] Le Guen V, Guyot J, Mattos CRR, Seguin M, Garcia D. 2008. Long lasting rubber tree resistance to *Microcyclus ulei* characterized by reduced conidial emission and absence of teleomorph. Crop Prot. 27(12):1498–1503.

[CIT0008] Lieberei R. 1986. Cyanogenesis of *Hevea brasiliensis* during infection with *Microcyclus ulei*. J Phytopathol. 115(2):134–146.

[CIT0009] Liu J, Shi C, Shi C-C, Li W, Zhang Q-J, Zhang Y, Li K, Lu H-F, Shi C, Zhu S-T, et al. 2020. The chromosome-based rubber tree genome provides new insights into spurge genome evolution and rubber biosynthesis. Mol Plant. 13(2):336–350. 31838037 10.1016/j.molp.2019.10.017

[CIT0010] Niu Y-F, Hu Y-S, Zheng C, Liu Z-Y, Liu J. 2020. The complete chloroplast genome of *Hevea camargoana*. Mitochondrial DNA B. 5(1):607–608. 10.1080/23802359.2019.1710605 PMC7748615 33366668

[CIT0011] Pootakham W, Sonthirod C, Naktang C, Ruang-Areerate P, Yoocha T, Sangsrakru D, Theerawattanasuk K, Rattanawong R, Lekawipat N, Tangphatsornruang S, et al. 2017. De novo hybrid assembly of the rubber tree genome reveals evidence of paleotetraploidy in *Hevea* species. Sci Rep. 7(1):41457. 28150702 10.1038/srep41457 PMC5288721

[CIT0012] Priyadarshan PM, Gonçalves PdS. 2002. Use of *Hevea* gene pool in rubber tree (*Hevea brasiliensis* Muell Arg.) breeding. The Planter. 78:123–138.

[CIT0013] Rahman AYA, Usharraj AO, Misra BB, Thottathil GP, Jayasekaran K, Feng Y, Hou S, Ong SY, Ng FL, Lee LS, et al. 2013. Draft genome sequence of the rubber tree *Hevea brasiliensis*. BMC Genomics. 14(1):75. 23375136 10.1186/1471-2164-14-75 PMC3575267

[CIT0014] Rocha ACS, Garcia D, Uetanabaro APT, Carneiro RTO, Araújo IS, Mattos CRR, Góes-Neto A. 2011. Foliar endophytic fungi from *Hevea brasiliensis* and their antagonism on *Microcyclus ulei*. Fungal Diver. 47(1):75–84.

[CIT0015] Tang C, Yang M, Fang Y, Luo Y, Gao S, Xiao X, An Z, Zhou B, Zhang B, Tan X, et al. 2016. The rubber tree genome reveals new insights into rubber production and species adaptation. Nat Plants. 2(6):16073. 2016.73. 27255837 10.1038/nplants.2016.73

[CIT0016] Tangphatsornruang S, Uthaipaisanwong P, Sangsrakru D, Chanprasert J, Yoocha T, Jomchai N, Tragoonrung S. 2011. Characterization of the complete chloroplast genome of *Hevea brasiliensis* reveals genome rearrangement, RNA editing sites and phylogenetic relationships. Gene. 475(2):104–112. 21241787 10.1016/j.gene.2011.01.002

[CIT0017] Wyman SK, Jansen RK, Boore JL. 2004. Automatic annotation of organellar genomes with DOGMA. Bioinformatics. 20(17):3252–3255. 15180927 10.1093/bioinformatics/bth352

